# SOD3 Reduces Inflammatory Cell Migration by Regulating Adhesion Molecule and Cytokine Expression

**DOI:** 10.1371/journal.pone.0005786

**Published:** 2009-06-04

**Authors:** Juha P. Laurila, Lilja E. Laatikainen, Maria D. Castellone, Mikko O. Laukkanen

**Affiliations:** 1 Medicity Research Laboratory, University of Turku, Turku, Finland; 2 Institute of Experimental Endocrinology and Oncology (CNR), Department of Biology and Cellular and Molecular Pathology, University of Naples Federico II, Naples, Italy; Tel Aviv University, Israel

## Abstract

Inflammatory cell migration characteristic of ischemic damages has a dual role providing the tissue with factors needed for tissue injury recovery simultaneously causing deleterious development depending on the quality and the quantity of infiltrated cells. Extracellular superoxide dismutase (SOD3) has been shown to have an anti-inflammatory role in ischemic injuries where it increases the recovery process by activating mitogen signal transduction and increasing cell proliferation. However, SOD3 derived effects on inflammatory cytokine and adhesion molecule expression, which would explain reduced inflammation in vascular lesions, has not been properly characterized. In the present work the effect of SOD3 on the inflammatory cell extravasation was studied *in vivo* in rat hind limb ischemia and mouse peritonitis models by identifying the migrated cells and analyzing SOD3-derived response on inflammatory cytokine and adhesion molecule expression. SOD3 overexpression significantly reduced TNFα, IL1α, IL6, MIP2, and MCP-1 cytokine and VCAM, ICAM, P-selectin, and E-selectin adhesion molecule expressions in injured tissues. Consequently the mononuclear cell, especially CD68+ monocyte and CD3+ T cell infiltration were significantly decreased whereas granulocyte migration was less affected. According to our data SOD3 has a selective anti-inflammatory role in ischemic damages preventing the migration of reactive oxygen producing monocyte/macrophages, which in excessive amounts could potentially further intensify the tissue injuries therefore suggesting potential for SOD3 in treatment of inflammatory disorders.

## Introduction

The inflammatory process is initiated by endothelial cell (EC) activation comprising upregulation of chemokines and cell adhesion molecules, leukocyte activation and transmigration, and secretion of proinflammatory factors by leukocytes [1]. The inflammatory reaction is necessary for tissue recovery as it provides the correct cytokine signals and cell machinery to clear up the site for regeneration of the tissue [2]. However, uncontrolled inflammation has unfavorable effects on the course of tissue healing since the inflammatory cells are also capable of inducing tissue damage [2] and therefore many conditions involving inflammation, e.g. autoimmune diseases and tissue transplantations, are treated with immunosuppressants to reduce harmful leukocyte infiltration. Among the most potent drugs are glucocorticoids that downregulate the expression of numerous inflammatory chemokines, cytokines and adhesion molecules [3]–[5], which, however, are not entirely without adverse effects such as delayed myocardial tissue healing, osteoporosis, and blood vessel calcification [6]–[9].

The most prominent outcome in the initial phase of inflammation is the enhanced production of cytokines, such as TNF-α and IL-1, and chemokines, such as monocyte chemoattractant protein-1 (MCP-1) [10], [11], which further induce expression of a number of inflammatory cytokines [4]. Many of the stimulus-specific pathways converge in the production of superoxide (O_2_^−^) and hydrogen peroxide (H_2_O_2_) as signal mediators, which in turn result in e.g. NFκB activation responsible for numerous stress-related functions [12]–[14]. Leukocytes are thus recruited by expression of various cell adhesion molecules, e.g. selectins, and intercellular and vascular cell adhesion molecules (ICAM-1 and VCAM-1, respectively) [15], [16]. They promote rolling and firm adhesion of leukocytes to endothelial wall, the necessary interactions preceding transmigration [17]. To aid leukocyte migration the vessel wall cells change their morphology by assuming cytoskeletal and cell-cell junction modifications in response to e.g. ligand binding to ICAM-1 and VCAM-1 [18]–[20], and when stimulated by O_2_^−^ or TNF-α [21], [22].

Previously, it has been shown that extracellular superoxide dismutase (SOD3) can attenuate tissue damage and inflammation but so far its mechanism of action has not been completely defined [23]–[27]. Since excess inflammation prevents the tissue injury recovery we investigated in the present study the effect of SOD3 overexpression on cell migration. We used two *in vivo* acute inflammation models to determine how SOD3 affects leukocyte extravasation, and compared the results to efficacy of the glucocorticoid immunosuppressant dexamethasone. The mouse peritonitis and rat hind limb ischemia models have been characterized previously: they induce rapid infiltration of leukocytes to the peritoneal cavity and large femoral muscles, respectively [28]–[30]. We then analyzed the proportions of the infiltrated leukocyte subtypes, and determined the effects on several mediators of the inflammatory reaction.

## Materials and Methods

### Ischemia model

Fischer 344 rats (Harlan, Horst, Netherlands) and Balb/C mice (local colony) were maintained in specific pathogen-free conditions and had access to food and water ad libitum. All experimental procedures were approved by the Experimental Animal Committee of University of Turku.

Ischemic hind limb injury was induced to male Fischer 344 rats (5 to 6 weeks old, 86–115 g) by surgical closure of the distal femoral artery, lateral circumflex femoral artery, and the proximal femoral artery. The animals were anesthetized for the procedure by intra peritoneal administration of fentanyl fluanisone (Janssen Pharmaceutica, Beerse, Belgium) and midazolame (Roche, Basel, Switzerland). Gene transfer was done immediately after the ligation by intra muscular injection of 0.5×10^9^ pfu adenovirus SOD3 (AdSOD3) or LacZ (AdLacZ) in 50 µl PBS as described [27], [31], [32]. Uninjured muscle tissue was used as control.

### Peritonitis model

Gene transfer was done to 8–10 week old female balb/c mice with intra peritoneal injection of 0.5×10^9^ pfu AdSOD3 or AdLacZ. Acute peritoneal inflammation was induced 72 hours later by i.p. injection of 1 ml PBS containing 5% proteose peptone (BD Difco, Sparks, MD, USA) and 10 ng of IL-1β (R&D Systems, Minneapolis, MN, USA). As a control treatment, animals were given 50 mg/kg of Dexamethasone (Oradexon, Organon, Oss, Holland) half an hour before proteose peptone injection. Cells from the peritoneal cavity were collected 18 hours after the induction of inflammation by washing with 10 ml of RPMI containing 5 U/ml heparin (Løvens Kemiske Fabrik, Ballerup, Denmark). Cells from peritoneal lavage were counted and cytocentrifuged at 1000 rpm for 5 minutes (Shandon cytospin 3, Shandon, Pittsburgh, PA, USA). Slides were stained with Reastain Diff-Quick (Reagena, Toivala, Finland) to analyze different leukocyte subtypes.

### Immunohistochemistry

Rat muscle samples were frozen in liquid nitrogen and embedded in Tissue-Tek optimal cutting temperature compound (Sakura Finetek, Torrance, CA, USA). Ten micrometer sections were fixed in acetone and stained with rabbit anti-rat CD3 and mouse anti-rat CD68 (Serotec, Oxford, UK). The sections were counterstained with hematoxylin/eosin (Sigma, Saint Louis, MI, USA). The number of CD3^+^ and CD68^+^ cells were analyzed from whole sections with Zeiss Axiovert 200 M (Carl Zeiss, Oberkochen, Germany).

### Reporter assay

HEK 293T cells were used for *in vitro* assay to provide a general cell model that we have previously used in our reporter, expression, and cell signalling studies allowing the comparison of the data with our previous results. HEK 293T cells were transfected with SOD3 expression vector together with pNFκB Luc reporter (Stratagene, Cedar Creek, TX, USA). Luciferase activity was quantified with Tecan Ultra XFluor4 Fluorescence Reader (Tekan, Mannedorf, Switzerlard).

### Western blot analysis

HEK293 cells were homogenized in lysis buffer (50 mmol/l HEPES pH 7.5, 150 mmol/l NaCl, 10% glycerol, 1% Triton X-100, 1 mmol/l MgCl, 10 mmol/l NaF, 10 mmol/l sodium pyrophosphate, 1 mmol/l Na_3_VO_4_, 10 µg/ml approtinin, 10 µg/ml leupeptin) (Sigma, Saint Louis, MI, USA). Mouse anti-human α-IκB (Santa Cruz, Santa Cruz, CA, USA) was used to detect IκB levels from tissues.

### Quantitative PCR

Total RNA was extracted from a pool of four animals using Tri-reagent (Sigma, Saint Louis, MI, USA). The first strand synthesis was done with Revert-Aid M-MuLV (Fermentas, Burlington, Canada), and the following quantitative PCR with SYBR Green PCR master mix (Applied Biosystems, Foster City, CA). Primers and cycling conditions are shown in the Table 1.

**Table 1:** Primers and cycling conditions.

Gene	Primer	Tm
TNFα for	AGA TGT GGA ACT GGC AGA GG	60
TNFα rev	CCC ATT TGG GAA CTT CTC CT	
IL-1α for	TCG GGA GGA GAC GAC TCT AA	58
IL-1α rev	GAA AGC TGC GGA TGT GAA GT	
IL-6 for	CCG GAG AGG AGA CTT CAC AG	55
IL-6 rev	ACA GTG CAT CAT CGC TGT TC	
MCP-1 for	CTC ACC TGC TGC TAC TCA TTC ACT	55
MCP-1 rev	TGC TGC TGG TGA TTC TCT TGT AGT	
MIP2 for	ATC CAG AGC TTG ACG GTG AC	55
MIP2 rev	GGA CTT GCC GCT CTT CAG TA	
ICAM for	AGG TAT CCA TCC ATC CCA CA	55
ICAM rev	GCC ACA GTT CTC AAA GCA CA	
VCAM for	TGA CAT CTC CCC TGG ATC TC	55
VCAM rev	CTC CAG TTT CCT TCG CTG AC	
PSEL for	TTC CCA CAC TTC CTT CTG CT	57
PSEL rev	CAC GCT GTA GTC GGG GTA TT	
ESEL for	TTT TTG GCA CGG TAT GTG AA	57
ESEL rev	AGG TTG CTG CCA CAG AGA GT	
β-actin for	TCG TGC GTG ACA TTA AGG AG	55
β-actin rev	GTC AGG CAG CTC GTA GCT CT	

### Statistical Analysis

All results are expressed as mean±SEM. A paired t-test was used to determine differences between groups.

## Results

### SOD3 inhibits leukocyte migration in acute ischemia

Neutrophils, macrophages, and other inflammatory cells mediate a number of important cellular functions in injured tissue [33], [34]. Phagocytotic macrophages clear cellular debris and secrete inflammatory cytokines such as MIP-2, a strong neutrophil attracting agent leading to further increase in inflammatory signaling [35]. However, excessive inflammatory reaction may also contribute to tissue damage by enhancing macrophage infiltration, which increases tissue free radical load leading to further tissue injury [36]. In addition, decreased neutrophil accumulation leads to reduced infarct size, reduced vascular permeability, and resistance in ischemia/reperfusion (I/R) injury [37], [38]. Thus, it is suggested that the tissue recovery is dependent on the factors regulating the inflammatory cell migration into the injuries. In the present work we studied leukocyte migration in acute ischemia and peritonitis models and analyzed the contribution of SOD3 on inflammatory cytokine and adhesion molecule expression.

**Figure 1:**
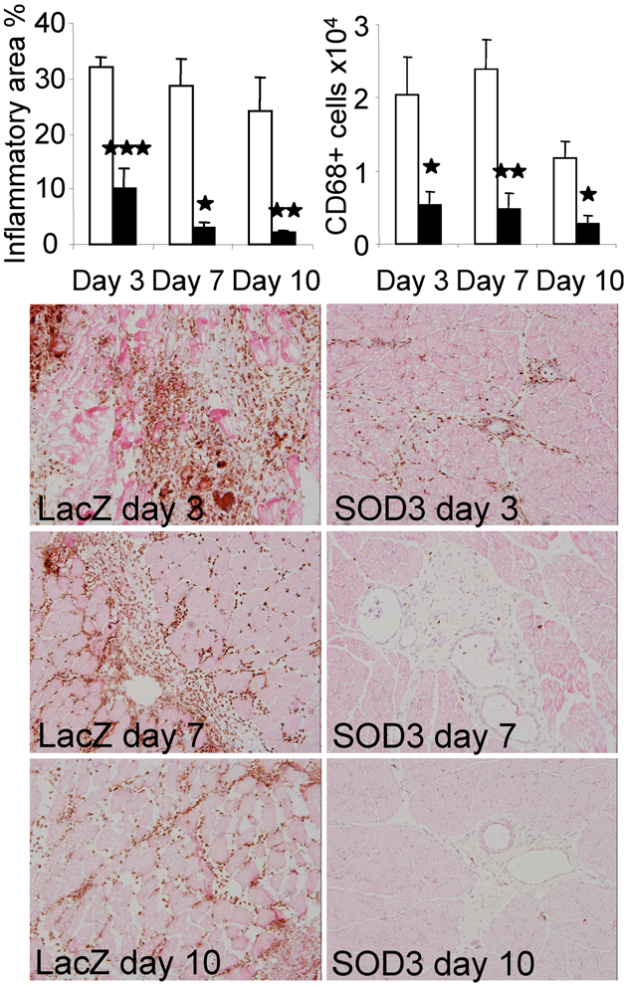
Reduced macrophage infiltration in to ischemic muscle. Open bars represent LacZ animals and black bars represent SOD3 animals. CD68 staining showed significantly reduced inflammatory area and macrophage infiltration in SOD3 animals at all time points studied. Histological stainings show CD68+ macrophages around the femoral artery in ischemic muscles (20× magnification).

We determined the effect of SOD3 overexpression on the degree of inflammation by analyzing the size of inflamed tissue and the number of infiltrated macrophages and T cells in acute ischemic injury model. In a mouse model of femoral artery ligation, macrophage infiltration into ischemic muscle reaches peak values 3 days after the injury [39]. Histological analysis of the rat hind limb ischemia showed 3-fold reduction in the inflamed tissue area as determined by the presence of CD68^+^ macrophages (p<0.001) in SOD3 vs. LacZ control animals 3 days after vessel ligation (Figure 1). The reduction became even more prominent in later time points reaching 12-fold difference 10 days after vessel ligation. Additionally, the number of infiltrated CD68^+^ macrophages was 3–5 fold higher in LacZ control animals as compared to SOD3 animals (p<0.05). Maximal macrophage accumulation to the injured tissue was seen at 7-day time point in LacZ animals indicating that the inflammatory reaction was still developing in control animals at the initial phase of the follow-up period while the inflammation was decreasing in SOD3 animals. Throughout the experiment the number of macrophages remained higher in control animals than initially observed in SOD3 animals, which by the 10-day time point showed values close to the background levels further underlining the beneficial effect of SOD3.

**Figure 2:**
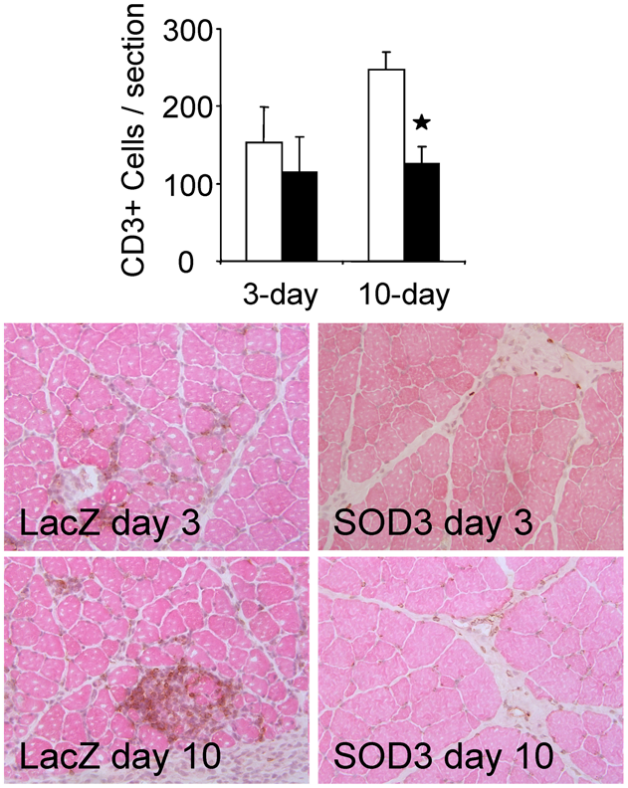
Inhibition of T-cell migration. Open bars represent LacZ animals and black bars represent SOD3 animals. Infiltration of CD3+ lymphocytes was inhibited in SOD3 animals 10 days after injury remaining at the level seen at earlier 3-day time point (20× magnification).

As compared to neutrophils and macrophages, the role of CD3^+^ T-cells in recovery of ischemic tissue has remained uncertain. Despite relatively low level of infiltration, studies suggest an early role for T-cells in attracting neutrophils and macrophages to site of myocardial or peripheral ischemia/reperfusion injury [40], [41]. The histological analysis at 3-day time point showed 114±23 and 153±46 (p = ns.) CD3^+^ T cells per ischemic tissue section in SOD3 and LacZ animals, respectively (Figure 2). At 10-day time point number of T cells had increased in LacZ animals by 60% to 247±31 whereas in SOD3 animals T cell accumulation remained at similar level as compared to 3 day time point (125±30, p<0.05). The analysis of leukocyte accumulation in the rat hind limb ischemia model shows selective inhibition of inflammatory cell migration and suggests SOD3 to have more prominent effect on macrophage infiltration as compared to lymphocytes.

### SOD3-mediated inhibition of leukocyte accumulation in peritonitis model

**Figure 3:**
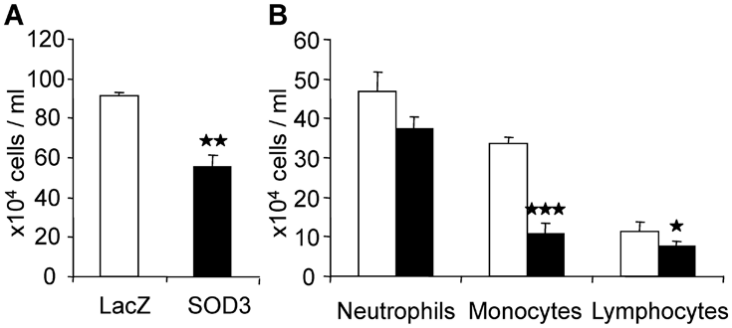
Anti-inflammatory effect of SOD3 affects predominantly macrophages in peritonitis model. Open bars represent LacZ animals and black bars represent SOD3 animals. (a) SOD3-mediated reduction in total leukocyte number in peritoneal lavage 18 hours after induction of inflammation. SOD3 treated animals had 55.6×10^4^ (±4.1) cells/milliliters of lavage as compared to 91.6×10^4^ (±6.7) found from LacZ control animals. (b) Analysis of different leukocyte subtypes showed strongest effect in macrophages although lymphocyte migration was also reduced. Monocyte accumulation in LacZ vs. SOD3 treatment was reduced from 33.5×10^4^ (±1.8) to 10.9×10^4^ (±2.5) cells/ml whereas lymphocytes were reduced from 11.4×10^4^ (±2.2) to 7.6×10^4^ (±1.2) cells/ml, and neutrophils from 47.0×10^4^ (±4.7) to 37.5×10^4^ (±2.9) cells/ml.

To confirm the findings and to further analyze the SOD3-derived selective inhibition of cell migration we examined leukocyte migration in a mouse peritonitis model, which provides an efficient way to analyze leukocyte traffic in an acute inflammatory response. To induce peritoneal inflammation we used 5% solution of proteose peptone supplemented with IL-1β and counted the numbers of different leukocyte subtypes from the peritoneal lavage 18 hours after induction of inflammation. The analysis of SOD3 overexpression derived inhibition of cell migration showed 20% (p = ns), 67% (p<0.001), and 33% (p<0.05) reduction in migrating neutrophils, monocytes/macrophages, and lymphocytes, respectively (Figure 3B). Moreover, the total number of infiltrated leukocytes was decreased by 30% in SOD3 animals as compared to LacZ controls (Figure 3A) (p<0.01), which is mostly caused by the effect of attenuated macrophage migration. The data effectively confirmed our findings in rat hind limb ischemia showing vastly stronger inhibition of macrophage infiltration as compared to other leukocyte subtypes.

**Figure 4:**
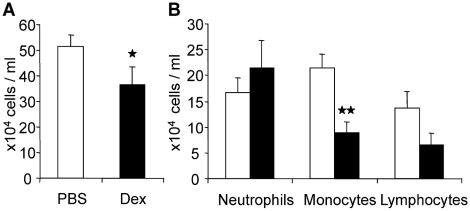
Anti-inflammatory effect of dexamethasone. Open bars represent PBS treated animals and black bars represent dexamethasone animals (dosage 50 mg/kg). (a) Leukocyte infiltration was reduced by dexamethasone treatment from 51.5×10^4^ (±4.7) to 36.5×10^4^ (±7.1) cells/milliliters of lavage. (b) Dexamethasone treatment had no effect on neutrophil migration, 16.6×10^4^ (±2.9) and 21.5×10^4^ (±5.2) cells/ml were found in PBS control group and dexamethasone treated animals, respectively. In contrast, accumulation of monocytes and lymphocytes were reduced from 21.4×10^4^ (±2.7) to 8.8×10^4^ (±2.2), and from 13.7×10^4^ (±3.2) to 6.5×10^4^ (±2.4), respectively.

Dexamethasone, a corticosteroid that reduces swelling and inflammation is a potent anti-inflammatory drug used to treat many bacteria-free inflammatory conditions, including rheumatoid arthritis and anaphylactic shock. Glucocorticoids exert their anti-inflammatory effect e.g. through repression of NF-κB mediated cytokine expression, which takes place after cytoplasmic glucocorticoid receptor translocates into the nucleus [5], [42]. To compare SOD3 to clinically approved medication we gave an intra-peritoneal injection of dexamethasone (Oradexon) to animals 30 minutes before induction of peritoneal inflammation. Leukocyte traffic to the inflamed peritoneum was reduced by 20% (p<0.05) after treatment with 50 mg/kg dexamethasone (Figure 4A). Monocyte/macrophage migration was reduced by 60% (p<0.01), and that of lymphocytes by marked 50% indicating tendency, while no significant difference was seen in neutrophil accumulation in this setting (Figure 4B). PBS mock treated animals exhibited lower neutrophil and monocyte accumulation as compared to animals subjected to LacZ gene transfer, which may be result of the adenovirus vector used in the study. Intriguingly, SOD3 treatment reduced peritoneal monocyte and lymphocyte numbers to similar level as dexamethasone treatment although neutrophil numbers remained higher than what was observed in either PBS or dexamethasone treated animals.

### SOD3 inhibits NF-κB activation and suppresses the inflammatory cytokine and adhesion molecule expression

**Figure 5:**
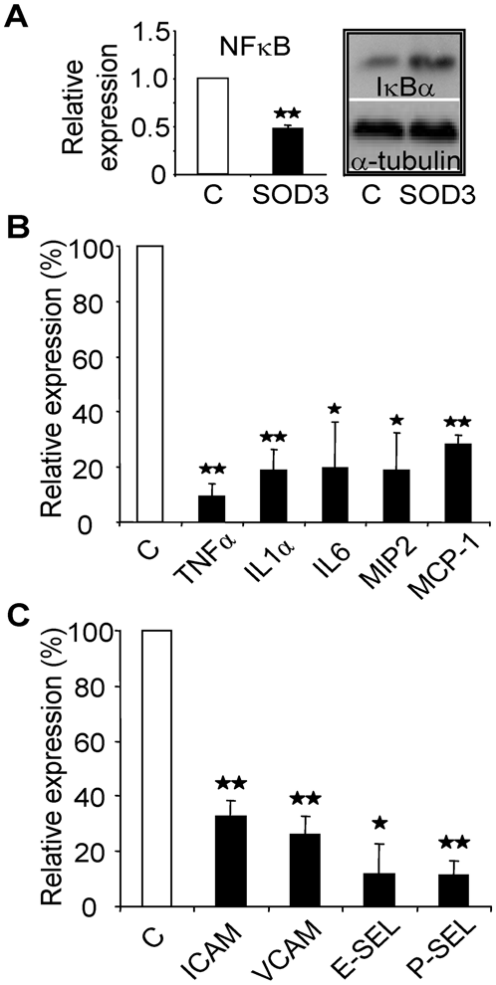
SOD3-mediated reduction in activity of inflammatory mediators. Open bars represent LacZ tissue and black bars represent SOD3 animals. (a) Luciferase assay shows 50% reduction in NF-κB activity *in vitro*, and western blot analysis shows increased IκBα expression. (b) Quantitative RT-PCR analysis for cytokines and chemokines. SOD3 overexpression derived reduced expression of TNFα, IL-1α, IL-6, MCP-1, and MIP2 in injured tissue. (b) Analysis of ICAM, VCAM, P-selectin, and E-selectin expression. Expression of inflammatory adhesion molecules was significantly reduced in SOD3 muscle.

Proinflammatory stimuli activate vascular endothelium leading to up-regulation of cell adhesion molecules and chemokines, NF-κB has been shown to be both necessary and sufficient for endothelial up-regulation of ICAM, VCAM, and MCP-1 [43]. Furthermore, ectopic expression of IκBα effectively abrogates expression of VCAM, IL-1, and IL-6 [44]. *In vitro* luciferase assay revealed 50% (p<0.01) decrease in NF-κB activity due to SOD3 transfection, which could at least partially be explained by increased IκBα expression (Figure 5A) suggesting that SOD3 promotes cytoplasmic localization of NF-κB rendering it incapable of binding DNA. NF-κB plays a central part in responses to inflammatory signaling by regulating the expression of cytokines suggesting that reduced NF-κB activity could lead to reduction in expression of inflammatory cytokines and chemokines. Therefore, we quantified cytokine and chemokine expression level *in vivo* from rat muscle three days after vessel ligation and SOD3 gene transfer. Quantitative RT-PCR showed significantly reduced expression of TNFα, IL1α, IL6, MIP2, and MCP1 (Figure 5B) in SOD3 animals suggesting reduced expression of several important inflammatory mediators. Specifically, MCP1 is an important macrophage attractant [45], [46], possibly explaining markedly reduced macrophage accumulation. Furthermore, since TNFα, IL1α, and IL6 are important regulators of endothelial adhesion molecule expression we analyzed expression of ICAM, VCAM, E-selectin, and P-selectin from the tissue (Figure 5C). We found significant reduction in adhesion molecule expression, which further confirms the reduction in overall inflammation in the muscle of SOD3 recipient rats as compared to LacZ control animals.

## Discussion

Tissue damage launches rapid recruitment of inflammatory leukocytes into injured tissue due to activation of endothelial cells. Inflammatory reaction promotes tissue healing by eliminating pathogens, clearing cellular debris, and promoting cell proliferation. However, excessive inflammatory reaction promotes injury e.g. through neutrophil-derived superoxide production [47]. In fact, reactive oxygen species function as inflammatory mediators by activating expression of cytokines such as TNFα, IL-1, and IL-6 [48] and therefore ROS may contribute to tissue injury by not only directly damaging the tissue but also by enhancing further leukocyte accumulation.

In the current work we showed that SOD3 is an important mediator of reduced CD68+ macrophage migration into the inflammatory area. Macrophages accumulate in high numbers to ischemic muscle forming the primary leukocyte population three days after injury [39]. CD68 staining showed significantly reduced inflammatory area and macrophage migration in SOD3 treated ischemic tissue as compared to LacZ control animals (Figure 1). The SOD3-mediated reduction in macrophage accumulation was evident in all of the studied time points. T-cells accumulate to ischemic muscle in vastly lower numbers as compared to macrophages. However, their presence is required for efficient neutrophil traffic, and they attract macrophages by secreting IL-16 [40]. In our studies, SOD3 did not prevent initial low level T-cell migration, but efficiently inhibited further increase at 10-day time point (Figure 2). Late effect on T-cell migration suggests an indirect mechanism for SOD3 mediated inhibition in T-cell traffic, which might be result of general decrease in inflammation. Inflammatory cytokines secreted by infiltrating macrophages attract other leukocytes to injured tissue [49]. Thereafter, inhibition of macrophage infiltration could lead to overall reduction in inflammatory reaction.

Since the SOD3-derived reduction in inflammation showed selective inhibition of macrophage migration, we were prompted to confirm the finding and to better characterize the cell specific effect. The mouse peritonitis model supported the SOD3-derived reduction in the number of infiltrating leukocytes (Figure 3A), which was predominantly due to reduced macrophage numbers (Figure 3B). These results confirm the anti-inflammatory property of SOD3 and show a stronger inhibition of monocyte migration as compared to other analyzed leukocyte subtypes. The data suggest that reduced superoxide tissue concentrations caused by SOD3 overexpression may explain the anti-inflammatory effect of the enzyme. It has been previously shown that superoxide treatment of rat cerebral endothelial cells increases monocyte adhesion and migration, which, however, was not replicated by H_2_O_2_ treatment but was instead abrogated by superoxide scavengers suggesting superoxide as an inflammatory mediator [21]. We have shown in our previous works that SOD3 overexpression *in vivo* efficiently decreases the production of superoxide in cardiovascular injuries including our hind limb ischemia model [25]–[27].

Anti-inflammatory medications currently available for clinical use include glucorticoid drugs such as dexamethasone. Dexamethasone binds the glucocorticoid receptor, which subsequently translocates to the nucleus and represses inflammatory gene expression by inhibiting e.g. NF-κB activity [4], [5], [42]. To compare the efficacy of SOD3 mediated anti-inflammatory effect to existing medication we determined the effect of Dexamethasone in mouse peritonitis. As a dose of 50 mg/kg, dexamethasone reduced leukocyte traffic in comparable levels to SOD3 gene transfer (Figure 4A). Dexamethasone-mediated effect was equally effective in monocyte/macrophage and lymphocyte lineages whereas no significant effect was seen in neutrophils (Figure 4B). Neutrophil accumulation has been shown to be at its highest as early as 4 hours after induction of inflammation in zymosan induced peritonitis [50]. Therefore, lack of effect on neutrophil migration could be due to late time point analyzed. The data suggests that SOD3 overexpression and dexamethasone administration have similar anti-inflammatory effect in acute inflammation and therefore suggesting SOD3 as a potential candidate molecule for clinical treatments.

NF-κB plays a crucial role in mediating inflammation due to its role in activating expression of pro-inflammatory genes such as cytokines TNFα and IL1α, and adhesion molecules ICAM-1 and VCAM-1 [43], [44]. Since NF-κB is a redox sensitive transcription factor being activated by oxidative stress, [12], [13] we analyzed the effect of SOD3 on NF-κB activity *in vitro* and showed significantly decreased activity. (Figure 5A). The data is in line with previous work in cardiovascular and liver transplantation models showing that increased NAPDH oxidase-derived superoxide production correlates with increased NF-κB activity, which is attenuated by SOD3 overexpression [51]–[54].

Since cytokines TNFα, IL1α, IL6, MIP2, and MCP1 are known to contain NF-κB binding sites in their gene promoters and are thus up-regulated by NF-κB activation [55]–[61], we analyzed their expression levels in rat muscle by quantitative PCR. All of the analyzed cytokines and chemokines were significantly down-regulated in SOD3 animals as compared to LacZ control animals (Figure 5B). TNFα, IL1α, and IL6 promote inflammatory cell migration by up-regulating E-selectin, P-selectin, ICAM, and VCAM. Furthermore, macrophage recruitment has been shown to be strongly dependent on MCP-1 secretion [46], while MIP2 is a strong attractant for neutrophils [35]. MCP-1 deficiency does not reduce the number of resident macrophages in peritoneal cavity, but prevents macrophage migration in response to acute thioglycollate induced peritonitis [46]. Lu et al. showed 3-fold reduction in macrophage migration in 2,4-dinitro-1-fluorobenzene induced skin hypersensitivity model while neutrophil numbers remained unchanged. Thereafter, marked down-regulation of MCP-1 seen in ischemic muscle could explain the observed strong macrophage inhibition. Finally, due to reduced inflammatory cytokine expression, we conducted further expression analyses and found reduced expression VCAM, ICAM, E-selectin, and P-selectin (Figure 5C). Reduced expression of common adhesion molecules highlights the anti-migratory role of SOD3. It has been shown that macrophage transmigration is strongly dependent on α_4_β_1_ integrin - ICAM-1 interaction. Pre-treatment of recipient mice before intra venous macrophage injection with monoclonal antibodies for ICAM-1 reduced macrophage migration to atherosclerotic plaques by 65% [62]. In addition, rolling and attachment of P388D1 mouse monocyte cell line was inhibited by P-selectin and VCAM antibodies in an *ex vivo* isolated perfused carotid artery model [63] demonstrating the importance of these adhesion molecules on macrophage transmigration.

In conclusion, our novel observation shows that SOD3 gene transfer into hind limb ischemia or peritonitis results in significantly reduced leukocyte migration due to decreased cytokine and adhesion molecule expression. Further on, the data suggest more pronounced anti-inflammatory effect on macrophages as compared to other leukocyte subtypes in the models used in the current work. The observed anti-inflammatory effect in SOD3 treated mice was comparable or even higher than that of Dexamethasone, which recently has been shown to have cardiovascular side effects [64], [65]. Our previous *in vivo* SOD3 overexpression models have suggested non-toxicity and beneficial effect on tissue protection and injury recovery [24]–[26], [31] suggesting that SOD3 overexpression by exogenous administration or through increased endogenous production in injured tissues could provide a promising medication against excess inflammatory cell migration.

## References

[1] Alom-Ruiz SP, Anilkumar N, Shah AM (2008). Reactive oxygen species and endothelial activation.. Antioxid Redox Signal.

[2] Tidball JG (2005). Inflammatory processes in muscle injury and repair.. Am J Physiol Regul Integr Comp Physiol.

[3] Rocksen D, Lilliehook B, Larsson R, Johansson T, Bucht A (2000). Differential anti-inflammatory and anti-oxidative effects of dexamethasone and N-acetylcysteine in endotoxin-induced lung inflammation.. Clin Exp Immunol.

[4] Nehme A, Edelman J (2008). Dexamethasone inhibits high glucose-, TNF-alpha-, and IL-1beta-induced secretion of inflammatory and angiogenic mediators from retinal microvascular pericytes.. Invest Ophthalmol Vis Sci.

[5] Barnes PJ (2006). Corticosteroid effects on cell signalling.. Eur Respir J.

[6] Bulkley BH, Roberts WC (1974). Steroid therapy during acute myocardial infarction. A cause of delayed healing and of ventricular aneurysm.. Am J Med.

[7] Weinstein RS, Jilka RL, Parfitt AM, Manolagas SC (1998). Inhibition of osteoblastogenesis and promotion of apoptosis of osteoblasts and osteocytes by glucocorticoids. Potential mechanisms of their deleterious effects on bone.. J Clin Invest.

[8] Kirton JP, Wilkinson FL, Canfield AE, Alexander MY (2006). Dexamethasone downregulates calcification-inhibitor molecules and accelerates osteogenic differentiation of vascular pericytes: implications for vascular calcification.. Circ Res.

[9] El-Helou V, Proulx C, Gosselin H, Clement R, Mimee A (2008). Dexamethasone treatment of post-MI rats attenuates sympathetic innervation of the infarct region.. J Appl Physiol.

[10] Ali MH, Schlidt SA, Chandel NS, Hynes KL, Schumacker PT (1999). Endothelial permeability and IL-6 production during hypoxia: role of ROS in signal transduction.. Am J Physiol.

[11] Liu X, He L, Stensaas L, Dinger B, Fidone S (2009). Adaptation to chronic hypoxia involves immune cell invasion and increased expression of inflammatory cytokines in rat carotid body.. Am J Physiol Lung Cell Mol Physiol.

[12] Schreck R, Rieber P, Baeuerle PA (1991). Reactive oxygen intermediates as apparently widely used messengers in the activation of the NF-kappa B transcription factor and HIV-1.. Embo J.

[13] Wei Z, Costa K, Al-Mehdi AB, Dodia C, Muzykantov V (1999). Simulated ischemia in flow-adapted endothelial cells leads to generation of reactive oxygen species and cell signaling.. Circ Res.

[14] Frey RS, Ushio-Fukai M, Malik A (2008). NADPH Oxidase-Dependent Signaling in Endothelial Cells: Role in Physiology and Pathophysiology.. Antioxid Redox Signal.

[15] Pueyo ME, Gonzalez W, Nicoletti A, Savoie F, Arnal JF (2000). Angiotensin II stimulates endothelial vascular cell adhesion molecule-1 via nuclear factor-kappaB activation induced by intracellular oxidative stress.. Arterioscler Thromb Vasc Biol.

[16] Chen XL, Zhang Q, Zhao R, Ding X, Tummala PE (2003). Rac1 and superoxide are required for the expression of cell adhesion molecules induced by tumor necrosis factor-alpha in endothelial cells.. J Pharmacol Exp Ther.

[17] Ley K, Laudanna C, Cybulsky MI, Nourshargh S (2007). Getting to the site of inflammation: the leukocyte adhesion cascade updated.. Nat Rev Immunol.

[18] Matheny HE, Deem TL, Cook-Mills JM (2000). Lymphocyte migration through monolayers of endothelial cell lines involves VCAM-1 signaling via endothelial cell NADPH oxidase.. J Immunol.

[19] Etienne-Manneville S, Manneville JB, Adamson P, Wilbourn B, Greenwood J (2000). ICAM-1-coupled cytoskeletal rearrangements and transendothelial lymphocyte migration involve intracellular calcium signaling in brain endothelial cell lines.. J Immunol.

[20] van Wetering S, van den Berk N, van Buul JD, Mul FP, Lommerse I (2003). VCAM-1-mediated Rac signaling controls endothelial cell-cell contacts and leukocyte transmigration.. Am J Physiol Cell Physiol.

[21] Van der Goes A, Wouters D, Van Der Pol SM, Huizinga R, Ronken E (2001). Reactive oxygen species enhance the migration of monocytes across the blood-brain barrier in vitro.. Faseb J.

[22] Gu Y, Xu YC, Wu RF, Souza RF, Nwariaku FE (2002). TNFalpha activates c-Jun amino terminal kinase through p47(phox).. Exp Cell Res.

[23] Folz RJ, Abushamaa AM, Suliman HB (1999). Extracellular superoxide dismutase in the airways of transgenic mice reduces inflammation and attenuates lung toxicity following hyperoxia.. J Clin Invest.

[24] Laukkanen MO, Leppanen P, Turunen P, Tuomisto T, Naarala J (2001). EC-SOD gene therapy reduces paracetamol-induced liver damage in mice.. J Gene Med.

[25] Laukkanen MO, Kivela A, Rissanen T, Rutanen J, Karkkainen MK (2002). Adenovirus-mediated extracellular superoxide dismutase gene therapy reduces neointima formation in balloon-denuded rabbit aorta.. Circulation.

[26] Brasen JH, Leppanen O, Inkala M, Heikura T, Levin M (2007). Extracellular superoxide dismutase accelerates endothelial recovery and inhibits in-stent restenosis in stented atherosclerotic Watanabe heritable hyperlipidemic rabbit aorta.. J Am Coll Cardiol.

[27] Laurila JP, Castellone MD, Curcio A, Laatikainen LE, Haaparanta-Solin M (2009). Extracellular superoxide dismutase is a growth regulatory mediator of tissue injury recovery.. Mol Ther.

[28] Merinen M, Irjala H, Salmi M, Jaakkola I, Hanninen A (2005). Vascular adhesion protein-1 is involved in both acute and chronic inflammation in the mouse.. Am J Pathol.

[29] Paterson IS, Klausner JM, Goldman G, Kobzik L, Welbourn R (1989). Thromboxane mediates the ischemia-induced neutrophil oxidative burst.. Surgery.

[30] Seekamp A, Ward PA (1993). Ischemia-reperfusion injury.. Agents Actions Suppl.

[31] Laukkanen MO, Lehtolainen P, Turunen P, Aittomaki S, Oikari P (2000). Rabbit extracellular superoxide dismutase: expression and effect on LDL oxidation.. Gene.

[32] Kozarsky KF, Wilson JM (1993). Gene therapy: adenovirus vectors.. Curr Opin Genet Dev.

[33] Fan J, Frey RS, Rahman A, Malik AB (2002). Role of neutrophil NADPH oxidase in the mechanism of tumor necrosis factor-alpha -induced NF-kappa B activation and intercellular adhesion molecule-1 expression in endothelial cells.. J Biol Chem.

[34] Gordon S (2003). Alternative activation of macrophages.. Nat Rev Immunol.

[35] Wolpe SD, Sherry B, Juers D, Davatelis G, Yurt RW (1989). Identification and characterization of macrophage inflammatory protein 2.. Proc Natl Acad Sci U S A.

[36] Ward PA, Warren JS, Johnson KJ (1988). Oxygen radicals, inflammation, and tissue injury.. Free Radic Biol Med.

[37] Jolly SR, Kane WJ, Hook BG, Abrams GD, Kunkel SL (1986). Reduction of myocardial infarct size by neutrophil depletion: effect of duration of occlusion.. Am Heart J.

[38] Korthuis RJ, Grisham MB, Granger DN (1988). Leukocyte depletion attenuates vascular injury in postischemic skeletal muscle.. Am J Physiol.

[39] Paoni NF, Peale F, Wang F, Errett-Baroncini C, Steinmetz H (2002). Time course of skeletal muscle repair and gene expression following acute hind limb ischemia in mice.. Physiol Genomics.

[40] Stabile E, Kinnaird T, la Sala A, Hanson SK, Watkins C (2006). CD8+ T lymphocytes regulate the arteriogenic response to ischemia by infiltrating the site of collateral vessel development and recruiting CD4+ mononuclear cells through the expression of interleukin-16.. Circulation.

[41] Yang Z, Day YJ, Toufektsian MC, Xu Y, Ramos SI (2006). Myocardial infarct-sparing effect of adenosine A2A receptor activation is due to its action on CD4+ T lymphocytes.. Circulation.

[42] Necela BM, Cidlowski JA (2004). Mechanisms of glucocorticoid receptor action in noninflammatory and inflammatory cells.. Proc Am Thorac Soc.

[43] Denk A, Goebeler M, Schmid S, Berberich I, Ritz O (2001). Activation of NF-kappa B via the Ikappa B kinase complex is both essential and sufficient for proinflammatory gene expression in primary endothelial cells.. J Biol Chem.

[44] Wrighton CJ, Hofer-Warbinek R, Moll T, Eytner R, Bach FH (1996). Inhibition of endothelial cell activation by adenovirus-mediated expression of I kappa B alpha, an inhibitor of the transcription factor NF-kappa B.. J Exp Med.

[45] Sica A, Wang JM, Colotta F, Dejana E, Mantovani A (1990). Monocyte chemotactic and activating factor gene expression induced in endothelial cells by IL-1 and tumor necrosis factor.. J Immunol.

[46] Lu B, Rutledge BJ, Gu L, Fiorillo J, Lukacs NW (1998). Abnormalities in monocyte recruitment and cytokine expression in monocyte chemoattractant protein 1-deficient mice.. J Exp Med.

[47] Entman ML, Youker K, Shoji T, Kukielka G, Shappell SB (1992). Neutrophil induced oxidative injury of cardiac myocytes. A compartmented system requiring CD11b/CD18-ICAM-1 adherence.. J Clin Invest.

[48] Nian M, Lee P, Khaper N, Liu P (2004). Inflammatory cytokines and postmyocardial infarction remodeling.. Circ Res.

[49] Cailhier JF, Partolina M, Vuthoori S, Wu S, Ko K (2005). Conditional macrophage ablation demonstrates that resident macrophages initiate acute peritoneal inflammation.. J Immunol.

[50] Getting SJ, Flower RJ, Perretti M (1997). Inhibition of neutrophil and monocyte recruitment by endogenous and exogenous lipocortin 1.. Br J Pharmacol.

[51] Azevedo LC, Pedro MA, Souza LC, de Souza HP, Janiszewski M (2000). Oxidative stress as a signaling mechanism of the vascular response to injury: the redox hypothesis of restenosis.. Cardiovasc Res.

[52] Hayashi T, Yamashita C, Matsumoto C, Kwak CJ, Fujii K (2008). Role of gp91phox-containing NADPH oxidase in left ventricular remodeling induced by intermittent hypoxic stress.. Am J Physiol Heart Circ Physiol.

[53] Lorne E, Zmijewski JW, Zhao X, Liu G, Tsuruta Y (2008). Role of extracellular superoxide in neutrophil activation: interactions between xanthine oxidase and TLR4 induce proinflammatory cytokine production.. Am J Physiol Cell Physiol.

[54] He SQ, Zhang YH, Venugopal SK, Dicus CW, Perez RV (2006). Delivery of antioxidative enzyme genes protects against ischemia/reperfusion-induced liver injury in mice.. Liver Transpl.

[55] Shakhov AN, Collart MA, Vassalli P, Nedospasov SA, Jongeneel CV (1990). Kappa B-type enhancers are involved in lipopolysaccharide-mediated transcriptional activation of the tumor necrosis factor alpha gene in primary macrophages.. J Exp Med.

[56] Collart MA, Baeuerle P, Vassalli P (1990). Regulation of tumor necrosis factor alpha transcription in macrophages: involvement of four kappa B-like motifs and of constitutive and inducible forms of NF-kappa B.. Mol Cell Biol.

[57] Mori N, Prager D (1996). Transactivation of the interleukin-1alpha promoter by human T-cell leukemia virus type I and type II Tax proteins.. Blood.

[58] Libermann TA, Baltimore D (1990). Activation of interleukin-6 gene expression through the NF-kappa B transcription factor.. Mol Cell Biol.

[59] Shimizu H, Mitomo K, Watanabe T, Okamoto S, Yamamoto K (1990). Involvement of a NF-kappa B-like transcription factor in the activation of the interleukin-6 gene by inflammatory lymphokines.. Mol Cell Biol.

[60] Widmer U, Manogue KR, Cerami A, Sherry B (1993). Genomic cloning and promoter analysis of macrophage inflammatory protein (MIP)-2, MIP-1 alpha, and MIP-1 beta, members of the chemokine superfamily of proinflammatory cytokines.. J Immunol.

[61] Ueda A, Ishigatsubo Y, Okubo T, Yoshimura T (1997). Transcriptional regulation of the human monocyte chemoattractant protein-1 gene. Cooperation of two NF-kappaB sites and NF-kappaB/Rel subunit specificity.. J Biol Chem.

[62] Patel SS, Thiagarajan R, Willerson JT, Yeh ET (1998). Inhibition of alpha4 integrin and ICAM-1 markedly attenuate macrophage homing to atherosclerotic plaques in ApoE-deficient mice.. Circulation.

[63] Ramos CL, Huo Y, Jung U, Ghosh S, Manka DR (1999). Direct demonstration of P-selectin- and VCAM-1-dependent mononuclear cell rolling in early atherosclerotic lesions of apolipoprotein E-deficient mice.. Circ Res.

[64] Evans N (1994). Cardiovascular effects of dexamethasone in the preterm infant.. Arch Dis Child Fetal Neonatal Ed.

[65] Bal MP, de Vries WB, van Oosterhout MF, Baan J, van der Wall EE (2008). Long-term cardiovascular effects of neonatal dexamethasone treatment: hemodynamic follow-up by left ventricular pressure-volume loops in rats.. J Appl Physiol.

